# The Impact of New-Onset Diabetes Mellitus and Hypertension on All-Cause Mortality in an Apparently Healthy Population: A Ten-Year Follow-Up Study

**DOI:** 10.1155/2021/3964013

**Published:** 2021-11-05

**Authors:** Suranut Charoensri, Kittrawee Kritmetapak, Tassanapong Tangpattanasiri, Chatlert Pongchaiyakul

**Affiliations:** ^1^Division of Endocrinology and Metabolism, Department of Medicine, Faculty of Medicine, Khon Kaen University, Khon Kaen, Thailand; ^2^Division of Nephrology, Department of Medicine, Faculty of Medicine, Khon Kaen University, Khon Kaen, Thailand; ^3^Department of Medicine, Faculty of Medicine, Khon Kaen University, Khon Kaen, Thailand

## Abstract

**Introduction:**

The comparative effect of new-onset diabetes mellitus (DM) and hypertension (HT) on long-term mortality is a matter of debate.

**Materials and Methods:**

From 2007 to 2017, a 10-year longitudinal retrospective cohort study was conducted in Thailand's tertiary care setting. As baseline data, health check-up data from apparently healthy participants without underlying disease from 2007 were extracted. The vital status of all participants was determined in 2017, ten years after an initial examination. The impact of new-onset DM and HT at baseline on 10-year all-cause mortality was investigated using multivariable logistic regression analysis.

**Results:**

The prevalence of new-onset DM and HT was 6.4% and 28.8%, respectively, at baseline. Newly diagnosed diabetes increased the risk of all-cause mortality over 10 years (adjusted OR 4.77 and 95% CI 2.23-9.99). HT, on the other hand, did not increase the risk of death (adjusted OR 1.24 and 95% CI 0.65-2.35). Different HT and DM status combinations were compared to a nondiabetic, nonhypertensive reference. Individuals who were diabetic and hypertensive had a greater risk of death (adjusted OR 6.22 and 95% CI 2.22-17.00). Having DM without HT also increased the risk of death (adjusted OR 4.36 and 95% CI 1.35-12.87). However, having HT without DM did not result in a significant increase in 10-year mortality risk (adjusted OR 1.21 and 95% CI 0.57-2.56).

**Conclusion:**

In an apparently healthy population, new-onset DM is more strongly associated with 10-year all-cause mortality than new-onset HT. Having both DM and HT was associated with a greater risk of death when compared to having DM or HT alone.

## 1. Background

Chronic noncommunicable diseases (NCDs), such as diabetes mellitus (DM) and hypertension (HT), are linked to an increased incidence of cardiovascular diseases (CVDs) [1], leading to an increase in all-cause mortality worldwide [2–5]. The relationship between these metabolic diseases and mortality varies by population, depending on a variety of factors (such as race/ethnicity, gender, and age, among others) [6–8]. Although DM and HT are not the leading causes of death in Thailand, public attention is drawn to these two diseases due to rising trends in prevalence among Thais and other Asian populations [9, 10]. Even in asymptomatic, new-onset patients, the presence of either DM or HT significantly increases the risk of atherosclerotic CVDs and death [11]. Previous studies found that patients with DM or HT had a higher mortality rate than the general population [12–14]. However, the comparative effect of both diseases on all-cause mortality remains unknown, particularly among Thais. As a result, the purpose of this study was to investigate the magnitude of new-onset DM and HT in predicting all-cause mortality after a 10-year follow-up in the Thai adult population.

## 2. Methods

### 2.1. Study Design and Participants

A 10-year hospital-based, retrospective cohort study was carried out at Srinagarind Hospital, a tertiary care facility in Thailand. Health check-up data from 2007 was extracted and defined as “baseline” data. The information included a complete medical history and laboratory results for 4,359 people who were apparently healthy and had not been diagnosed with any underlying diseases. After being informed about the study protocol, all subjects signed a written consent form at the start of the study. At their baseline examination, they were diagnosed with DM and/or HT. In 2017, ten years after the initial evaluation, all participants' vital status was determined using outpatient medical records, electronic medical records, telephone, or personal contact. The incidence of new-onset DM and HT over a 10-year period was also examined. Participants with missing baseline variable data (*N* = 92), missing 10-year follow-up data (*N* = 2,511), or new-onset DM and/or HT after baseline recruitment (*N* = 539) were excluded, leaving 1,217 in the final analysis. Participants were divided into four groups based on their baseline diagnosis of HT and DM status: (i) those who were free of both HT and DM (HT (−)/DM (−)), (ii) nondiabetic hypertensive individuals (HT (+)/DM (−)), (iii) diabetic individuals without hypertension (HT (−)/DM (+)), and (iv) diabetic hypertensive individuals (HT (+)/DM (+)). All research protocols were carried out in accordance with applicable guidelines and regulations. The Human Research Ethics Committee of Khon Kaen University reviewed and approved the study per the Helsinki Declaration and the Good Clinical Practice Guidelines (HE611278).

### 2.2. Anthropometric Measurements

The health check-up procedure included a thorough history-taking and physical examination of all participants. The participant's age was determined by calculating the date of his or her birthday. Each participant's body weight (BW) and height were measured using an electronic balance (accuracy 0.1 kg) and a stadiometer (nearest 0.1 cm), respectively. Body mass index (BMI) was calculated by dividing weight (kg) by height^2^ (m^2^). After the participants had been seated and rested for at least 5 minutes, their systolic and diastolic blood pressures (SBP and DBP) were measured twice using a standardized sphygmomanometer. The average of the two measurements was then used for all analyses.

### 2.3. Laboratory Measurements

Serum samples were collected in the morning after participants had fasted for 8-12 hours and immediately centrifuged. Fasting plasma glucose (FPG), serum creatinine (Cr), total cholesterol (TC), and triglyceride (TG) levels were all measured. The glucose oxidase method was used in this study for FPG, and enzymatic methods with an automatic autoanalyzer (Cobas Integra 800; Roche Diagnostics, Mannheim, Germany) were used for serum Cr, TC, and TG. The Chronic Kidney Disease Epidemiology Collaboration (CKD-EPI) equation was used to calculate the estimated glomerular filtration rate (eGFR) [15].

### 2.4. Operational Definition at Baseline

HT was classified as SBP of more than 140 mmHg and/or DBP of more than 90 mmHg according to The Seventh Report of the Joint National Committee on Prevention, Detection, Evaluation, and Treatment of High Blood Pressure (JNC7) guideline [16]. Participants were diagnosed with DM if their fasting plasma glucose (FPG) ≥ 126 mg/dL according to the 2010 American Diabetes Association (ADA) guideline [17]. FPG was repeated within one week in participants with abnormal results to confirm the diagnosis.

### 2.5. Statistical Analysis

All statistical analyses were performed using R (de Micheaux, Drouilhet, & Liquet, 2014). Data were presented as median and interquartile range (IQR) and proportions for continuous and categorical variables, respectively. Wilcoxon rank-sum test, Chi-square, and Fisher's exact test were used to calculate the *P* value as appropriate. Univariable logistic regression analysis was performed to calculate the odds ratios (ORs) and 95% confidence interval (95% CI) between each metabolic parameter and 10-year all-cause mortality. Multivariable logistic regression was used to assess confounding and effect modification from other potential risks. The variables with a *P* value <0.20 from the univariable analysis were entered into a multiple logistic regression model. *P* value <0.05 was considered statistically significant.

## 3. Results

A total of 1,217 participants (421 men and 796 women) were recruited in the final analysis. At the start of the study, the median age was 46 years (IQR 41-53). Participants were slightly overweight overall, according to the World Health Organization (WHO) Asia-Pacific criteria [18], with a median BMI of 23.2 kg/m^2^ (IQR 21.2-25.6). The prevalence of new-onset DM and HT was 6.4% and 28.8%, respectively, at baseline. Forty-two participants (3.5%) had both DM and HT (Table 1). There were 41 men (52.6%) men and 37 women (47.4%) among the DM participants. At the time of DM diagnosis, the median age and BMI were 54 years old (IQR 44-60) and 26.1 kg/m^2^ (IQR 23.9-28.6), respectively. There were 171 men (48.7%) and 180 women (51.3%) among the HT participants. At the time of HT diagnosis, the median age and BMI were 52 years old (IQR 45-59) and 25.0 kg/m^2^ (IQR 22.9-27.6), respectively.

In this study, 54 (4.4%) died from all causes during the 10-year follow-up period, with 29 (53.7%) men and 25 (46.3%) women. Nonsurvivors were on average older, had higher BMI, FPG, SBP, DBP, TG, and lower eGFR than survivors, while TC did not differ. When compared to the survivors at baseline, nonsurvivors had a higher prevalence of DM (29.6% vs. 5.3%) and HT (55.6% vs. 27.6%) (Table 1).

**Figure 1:**
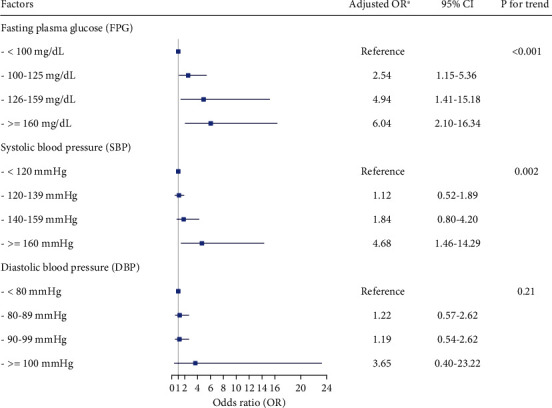
Associations of fasting plasma glucose (FPG), systolic blood pressure (SBP), and diastolic blood pressure (DBP) after subgroup categorization for the severity with all-cause mortality during the 10-year follow-up. ^∗^Model consisted of sex, age, BMI, eGFR, and TG at baseline. *P* value <0.05 was considered statistically significant. OR: odds ratio; BMI: body mass index; eGFR: estimated glomerular filtration rate; TG: triglyceride; DM: diabetes mellitus; HT: hypertension.

In univariate analysis, male sex, age, BMI, FPG, SBP, DBP, eGFR, and TG were associated with 10-year all-cause mortality. After adjusting for sex, age, BMI, FPG, SBP, DBP, eGFR, and TG, we found that old age, elevated FPG, and SBP remained as significant predictors of mortality. The adjusted odds ratios (ORs) with 95% CI were 1.12 (1.08-1.16), 1.16 (1.07-1.27), and 1.31 (1.06-1.63) for age per 1-year increase, FPG per 10 mg/dL increase, and SBP per 10 mmHg increase, respectively (Table 2). When categorized FPG, SBP, and DBP into groups based on level, elevated FPG and SBP were significantly associated with an increased risk of death, independent of other baseline parameters. However, there was no association between DBP and mortality in the study (Figure 1).

**Figure 2:**
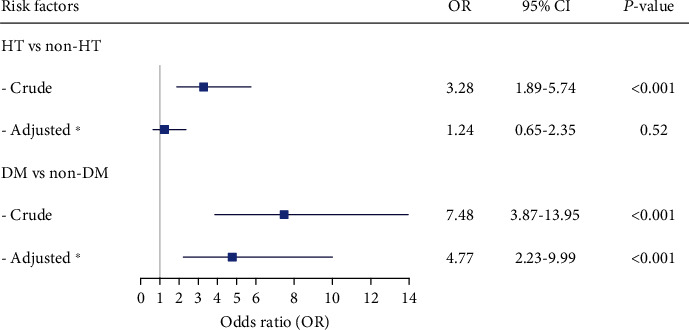
Association of new-onset HT and new-onset DM at baseline with all-cause mortality after 10-year follow-up. ^∗^Model consisted of sex, age, BMI, eGFR, TG, HT at baseline, and DM at baseline. *P* value <0.05 was considered statistically significant. DM: diabetes mellitus; HT: hypertension.

New-onset HT and DM at baseline increased the risk of 10-year all-cause mortality in an unadjusted model, with OR of 3.28 (95% CI 1.89-5.74) and 7.48 (95% CI 3.87-13.95) for HT and DM, respectively. The association of DM and all-cause mortality remained unchanged after adjusting sex, age, BMI, eGFR, TG, HT at baseline, and DM at baseline, with an adjusted OR of 4.77 (95% CI 2.23-9.99). However, HT did not increase the risk of mortality after adjusting (OR 1.24 and 95% CI 0.65-2.35) (Figure 2).

**Figure 3:**
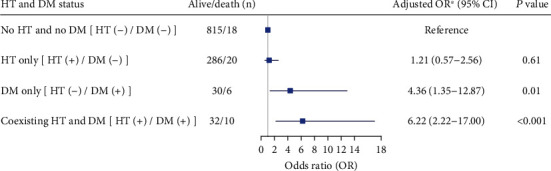
Multivariable logistic regression of different combinations of hypertension (HT) and diabetes mellitus (DM) status in association with all-cause mortality after 10-year follow-up. ^∗^Model adjusted with sex, age, BMI, eGFR, and TG at baseline. *P* value <0.05 was considered statistically significant. DM: diabetes mellitus; HT: hypertension.

The multivariable analysis of 10-year all-cause mortality was performed in the 3 categories of different combinations of HT and DM status compared to the reference (HT (−)/DM (−)). We found that diabetic hypertensive individuals (HT (+)/DM (+)) had a greater risk of death, with an adjusted OR of 6.22 (95% CI 2.22-17.00), and diabetic without hypertension (HT (−)/DM (+)) also increased the risk of death (adjusted OR 4.36 and 95% CI 1.35-12.87). However, being nondiabetic hypertensive individuals (HT (+)/DM (-)) did not show significant association with an increased risk of 10-year mortality (adjusted OR 1.21 and 95% CI 0.57-2.56) (Figure 3).

## 4. Discussion

The study's main findings revealed that in an apparently healthy general population, new-onset DM had a greater impact on 10-year all-cause mortality than new-onset HT. After adjusting for other metabolic risk factors including sex, age, BMI, eGFR, and TG level, having diabetes alone or in combination with HT increased the risk of all-cause mortality compared to those who were HT (-)/DM (-). Furthermore, we found that higher levels of FPG and SBP were independent predictors of 10-year mortality, whereas DBP had no such relationship.

Many previous studies have demonstrated the independent impact of hypertension and diabetes on all-cause and CVD mortality [19–22]. Furthermore, many studies have compared the impact of various combinations of HT and DM status on CVD and CVD mortality, using HT (-)/DM (-) as a reference. In some, but not all prospective studies, having both diseases or having DM alone had a greater impact on CVD events and mortality than having HT alone, similar to our findings [23–25]. A large cohort study conducted in Finland found that individuals with DM at baseline had a higher risk of coronary artery disease, stroke, and CVD mortalities when compared to HT [23, 24]. However, Oh et al. found that HT was more strongly associated with all-cause and CVD mortality than DM in people over the age of 55 [26]. These contradictory results could be attributed to differences in the study population's characteristics, such as age and ethnicity.

Sritara et al. conducted a similar study on 3,499 Thai employees of the Electricity Generating Authority of Thailand (EGAT) and found that age, SBP, DBP, smoking, and diabetes were all associated with vascular mortality [27]. The comparative effect of HT and DM on all-cause mortality, on the other hand, was not identified in this study. We extended a previous study in Thais by demonstrating that people with both HT and DM had a greater risk of dying from any cause; being HT (-)/DM (+) had a greater impact on death than being HT (+)/DM (-). This is significant, given that a higher proportion of DM patients in Thailand remain undiagnosed when compared to HT patients [28–30]. As a result, early detection of diabetes, followed by appropriate intervention, should be implemented to avoid long-term complications and mortality.

Several hypotheses could explain why diabetic patients have a higher risk of death. Diabetes, as we know, is a chronic disease with multiple etiologies characterized by abnormal glucose tolerance and carbohydrate metabolism due to defective insulin secretion, insulin resistance, or both [31]. Chronic hyperglycemia in people with diabetes is linked to an increase in the production of glycation end products and superoxide free radicals, which leads to diabetic angiopathy, endothelial dysfunction, inflammation, and cellular damage [32]. These conditions significantly increase the risk of atherosclerotic cardiovascular disease and death. Nonetheless, diabetes is linked to a higher risk of death from noncardiovascular diseases such as malignant neoplasms, infections, and even external causes of death [2, 33]. The mechanism for the diabetes-cancer link has been hypothesized to be primarily related to hyperinsulinemia, inflammation, and even certain treatments that may promote carcinogenesis [34]. Furthermore, chronic hyperglycemia in DM is thought to cause immunological dysfunction, resulting in a failure to control pathogen penetration in DM subjects. As a result, diabetic subjects are known to be more susceptible to infections [35]. On the contrary, HT is primarily associated with atherosclerotic cardiovascular death, which is caused by increased vasculature stiffness, neurohormonal dysregulation, and mechanical stress, resulting in cellular injury [5, 36].

In this study, we found that FPG, which is a metabolic parameter of DM, was a significant predictor of 10-year all-cause mortality after controlling for other variables. SBP also showed an association with mortality. However, this relationship was evident only in severe hypertensive range (≥160 mmHg), which may explain why the diagnosis of hypertension did not show association with mortality in our study. Pooled analysis of many observational studies found a linear increasing association of SBP to mortality, which is consistent with our findings [20]. However, in our study, we found that increasing DBP had no significant association with mortality. This could be explained by the different effects of DBP on different subgroups. Some studies found an increased association between DBP and all-cause and cardiovascular mortality in younger participants but not in elderly participants [37, 38]. This DBP phenomenon may be related to the progressive stiffening of the elastic arteries as we age. Low diastolic blood pressure in these elderly patients may precipitate coronary events and increase mortality [38].

The strengths of our study include a 10-year follow-up period and data collection in a registered health check-up database, which minimized information bias. Furthermore, we excluded patients who were diagnosed with new-onset HT and/or DM after baseline recruitment, which helped ensure that the association was completely related to the HT and DM status at baseline. However, the findings should be interpreted in the context of some potential limitations. First, we lack information on other clinical risk factors such as smoking status, alcohol consumption, dietary habits, and amount of exercise, all of which could influence mortality. Second, no follow-up information for any of the metabolic profiles was collected over a 10-year period. Because metabolic parameters such as SBP, DBP, FPG, eGFR, and lipid profiles can change over time, a lack of data addressing these changes may jeopardize the validity of our findings. Nonetheless, our study design reflects a real-life health check-up scenario in which patients only visited the clinic once, only a single clinical and laboratory assessment was performed, and follow-up data were sometimes unavailable. Third, our study uses only fasting glucose as the diagnosis criteria for DM, but in Asian population, abnormal postprandial glucose could be a major clinical phenotype [39]. When only use abnormality of fasting glucose to diagnose DM, a lot of diabetic patients may be missed. Finally, during the 10-year follow-up period, all-cause mortality occurred in only 4.4% of participants, indicating that our cohorts were “low-risk” patients. However, because of the small number of deaths, the effect of DM and HT on mortality may be imprecise.

In conclusion, new-onset DM is more strongly associated with 10-year all-cause mortality than new-onset HT in an apparently healthy population. A greater risk was shown when DM accompanied by HT compared with the DM alone or HT alone. As a result, early detection of DM is important to prevent long-term mortality in Thai population. More studies are required to assess the impact of HT versus DM on CVD events and all-cause mortality in other ethnicities.

## Figures and Tables

**Table 1:** Baseline characteristics of study participants according to 10-year vital status.

	Total (*n* = 1,217)	Alive (*n* = 1,163)	Death (*n* = 54)	*P* value
Male sex, *n* (%)	421 (34.6)	392 (33.7)	29 (53.7)	0.003^∗^
Age at baseline, years	46 (41, 53)	45 (40, 52)	61 (54, 66)	<0.001^∗^
BMI, kg/m^2^	23.2 (21.2, 25.6)	23.1 (21.1, 25.4)	24.9 (22.3, 27.1)	0.002^∗^
FPG, mg/dL	85 (79, 93)	85 (79, 92)	97 (87, 121)	<0.001^∗^
SBP, mmHg	118 (110, 130)	117 (110, 130)	134 (116, 152)	<0.001^∗^
DBP, mmHg	81 (73, 88)	80 (73, 88)	88 (79, 93)	<0.001^∗^
eGFR, mL/min/1.73 m^2^	95.1 (81.4, 107.2)	95.1 (82.2, 107.2)	80.6 (68.1, 97.0)	<0.001^∗^
Total cholesterol, mg/dL	209 (180, 235)	208 (180, 235)	217 (187, 239)	0.16
Triglyceride, mg/dL	103 (68, 151)	102 (68,150)	118 (88, 181)	0.01^∗^
Diagnosis of DM, *n* (%)	78 (6.4)	62 (5.3)	16 (29.6)	<0.001^∗^
Diagnosis of HT, *n* (%)	351 (28.8)	321 (27.6)	30 (55.6)	<0.001^∗^
Diagnosis of DM and HT, *n* (%)	42 (3.5)	32 (2.8)	10 (18.5)	<0.001^∗^

Data are presented as median and interquartile range (IQR 25-75%) and proportions for continuous and categorical variables, respectively. Wilcoxon rank-sum test, Chi-square, and Fisher's exact test were used to calculate the *P* value as appropriate. ^∗^*P* < 0.05 was considered statistically significant. BMI: body mass index; SBP: systolic blood pressure; DBP: diastolic blood pressure; FPG: fasting plasma glucose; eGFR: estimated glomerular filtration rate; DM: diabetes mellitus; HT: hypertension.

**Table 2:** Univariable and multivariable logistic regression analysis of baseline metabolic parameters in prediction of all-cause mortality during the 10-year follow-up.

	10-year all-cause mortality			
	Crude OR (95% CI)	*P* value	Adjusted OR (95% CI)	*P* value
Male sex	2.28 (1.32-3.97)	0.003^∗^	1.63 (0.86-3.10)	0.13
Age, per 1-year increase	1.14 (1.11-1.18)	<0.001^∗^	1.12 (1.08-1.17)	<0.001^∗^
BMI, per 1 kg/m^2^ increase	1.08 (1.01-1.16)	0.02^∗^	0.99 (0.90-1.08)	0.84
FPG, per 10 mg/dL increase	1.19 (1.12-1.27)	<0.001^∗^	1.17 (1.08-1.27)	<0.001^∗^
SBP, per 10 mmHg increase	1.56 (1.37-1.79)	<0.001^∗^	1.42 (1.12-1.79)	0.003^∗^
DBP, per 10 mmHg increase	1.55 (1.23-1.95)	<0.001^∗^	0.82 (0.55-1.20)	0.31
eGFR, per 10 mL/min/1.73 m^2^ decrease	1.08 (1.21-1.52)	<0.001^∗^	1.02 (0.84-1.24)	0.51
TC, per 10 mg/dL increase	1.03 (0.97-1.10)	0.28	-	-
TG, per 10 mg/dL increase	1.02 (1.00-1.04)	0.03^∗^	0.99 (0.95-1.02)	0.54

^∗^ *P* < 0.05 was considered statistically significant. OR: odds ratio; BMI: body mass index; SBP: systolic blood pressure; DBP: diastolic blood pressure; FPG: fasting plasma glucose; eGFR: estimated glomerular filtration rate; TC: total cholesterol; TG: triglyceride.

## Data Availability

Data are available on request from authors.
